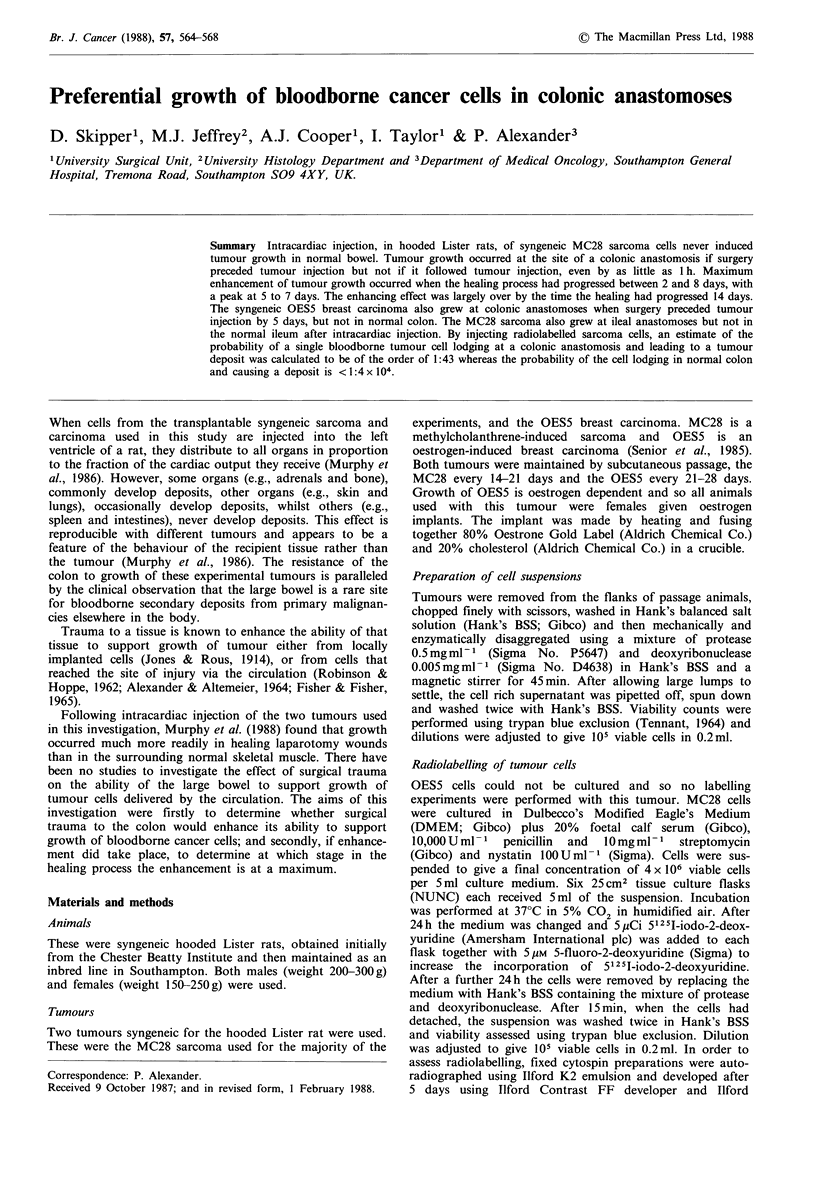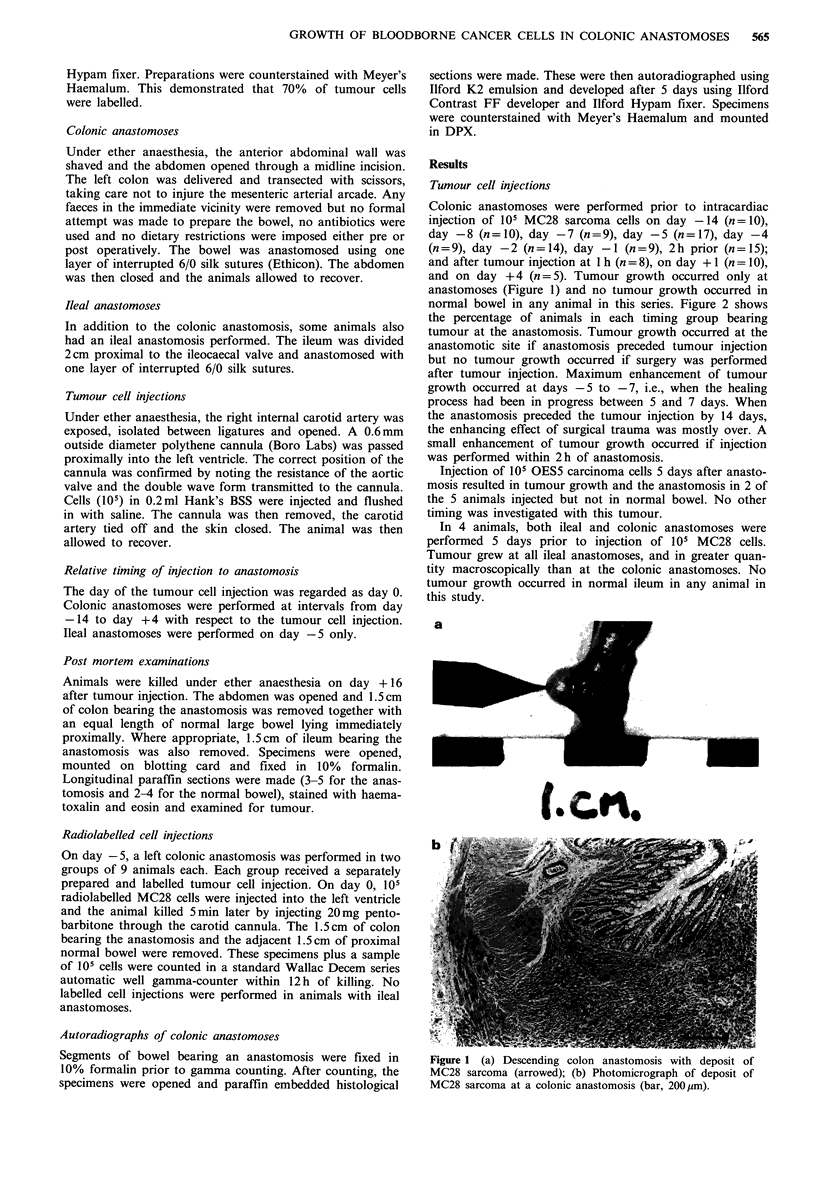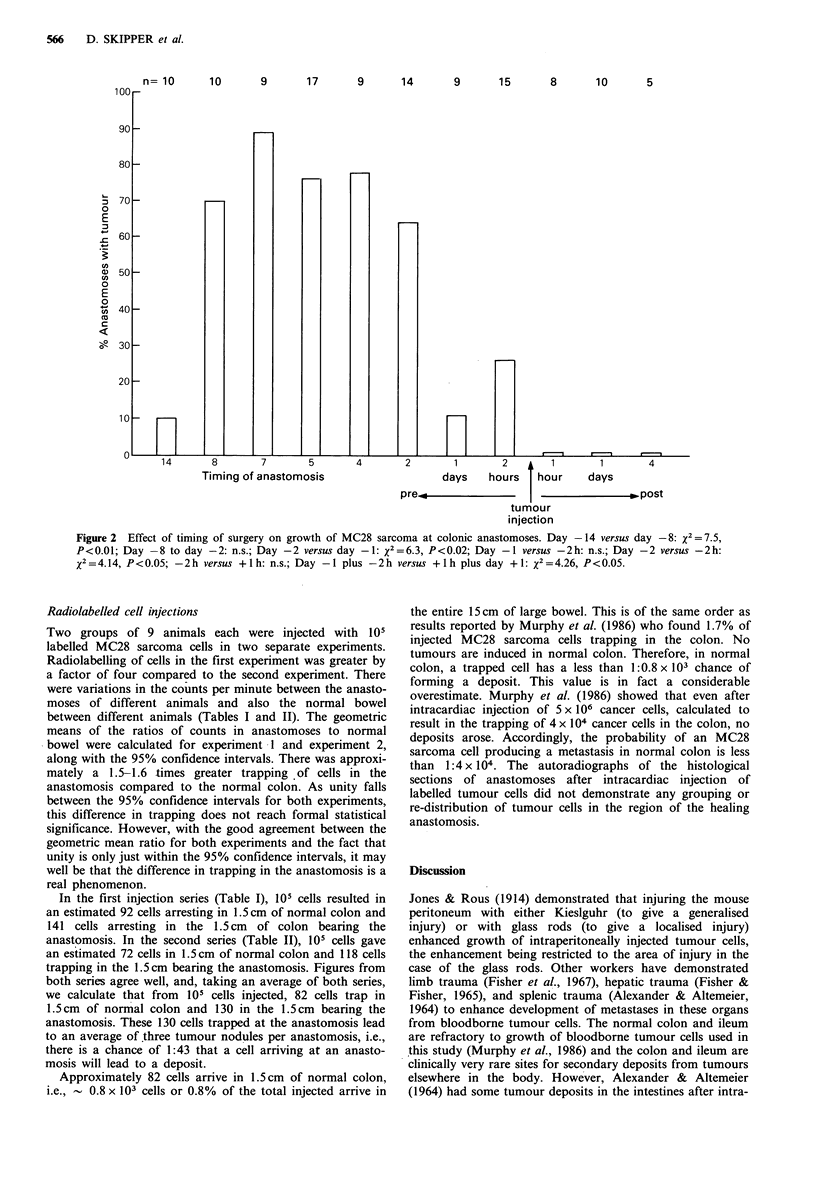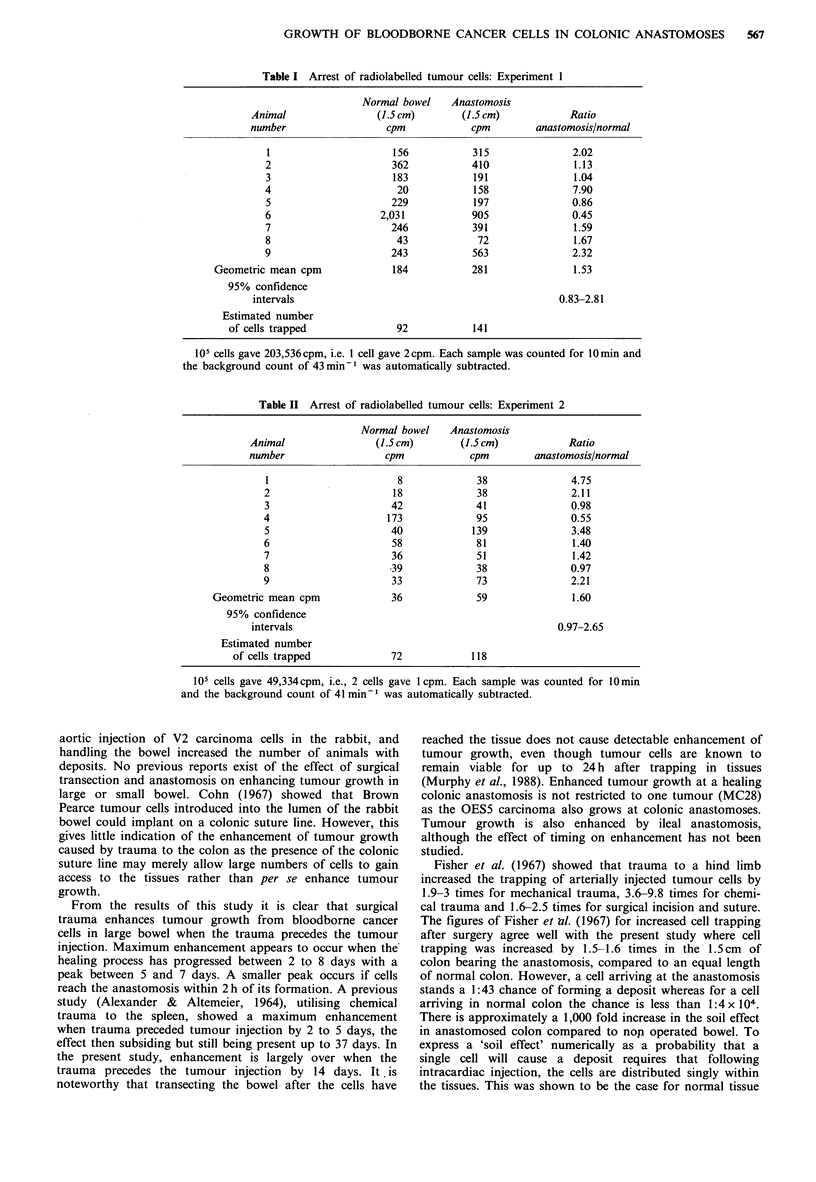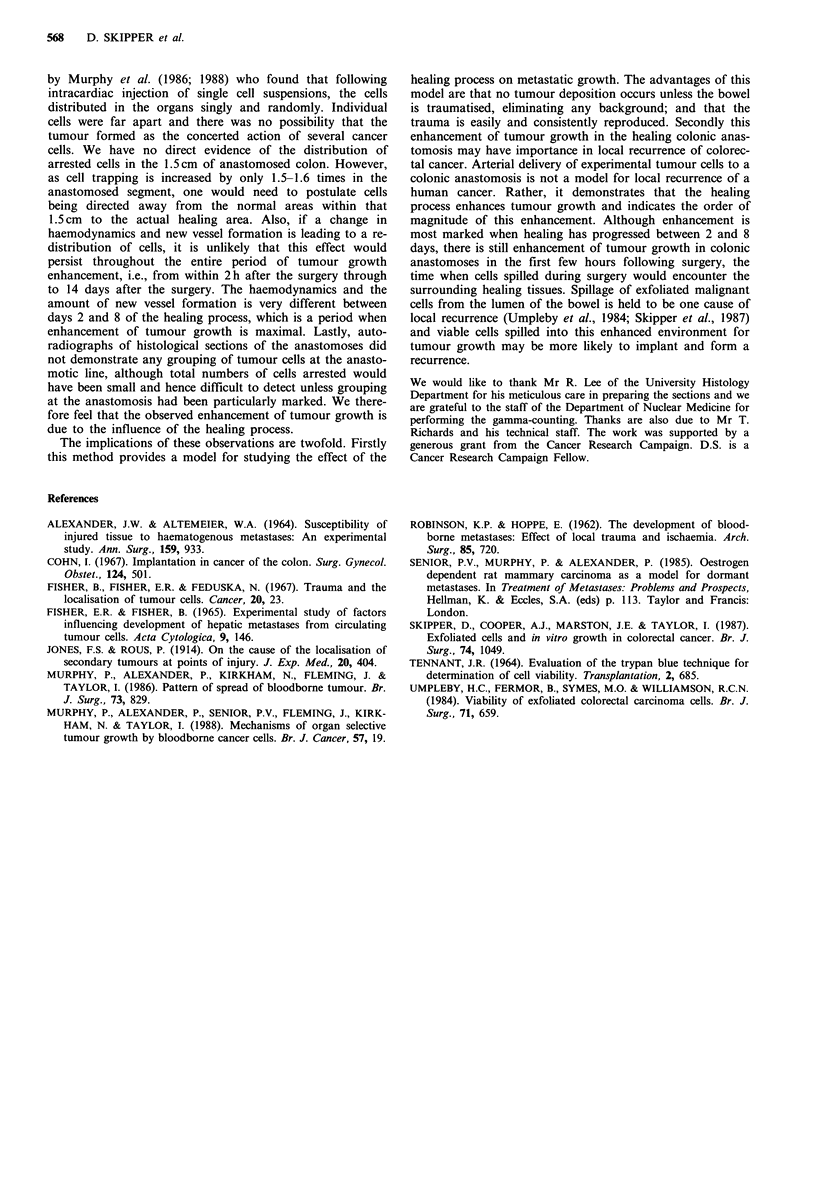# Preferential growth of bloodborne cancer cells in colonic anastomoses.

**DOI:** 10.1038/bjc.1988.129

**Published:** 1988-06

**Authors:** D. Skipper, M. J. Jeffrey, A. J. Cooper, I. Taylor, P. Alexander

**Affiliations:** University Surgical Unit, Southampton General Hospital, UK.

## Abstract

**Images:**


 © The Macmillan Press Ltd, 1988

### Preferential growth of bloodborne cancer cells in colonic anastomoses

D. Skipper[1], M.J. Jeffrey[2], A.J. Cooper[1], I. Taylor[1] & P. Alexander[3]

[1]*University Surgical Unit,* [2]*University Histology Department and* [3]*Department of Medical Oncology, Southampton General Hospital, Tremona Road, Southampton SO9 4XY, UK.*

**Summary** Intracardiac injection, in hooded Lister rats, of syngeneic MC28 sarcoma cells never induced tumour growth in normal bowel. Tumour growth occurred at the site of a colonic anastomosis if surgery preceded tumour injection but not if it followed tumour injection, even by as little as 1 h. Maximum enhancement of tumour growth occurred when the healing process had progressed between 2 and 8 days, with a peak at 5 to 7 days. The enhancing effect was largely over by the time the healing had progressed 14 days. The syngeneic OES5 breast carcinoma also grew at colonic anastomoses when surgery preceded tumour injection by 5 days, but not in normal colon. The MC28 sarcoma also grew at ileal anastomoses but not in the normal ileum after intracardiac injection. By injecting radiolabelled sarcoma cells, an estimate of the probability of a single bloodborne tumour cell lodging at a colonic anastomosis and leading to a tumour deposit was calculated to be of the order of 1:43 whereas the probability of the cell lodging in normal colon and causing a deposit is $<1:4\times10^4$.

When cells from the transplantable syngeneic sarcoma and carcinoma used in this study are injected into the left ventricle of a rat, they distribute to all organs in proportion to the fraction of the cardiac output they receive (Murphy *et al.*, 1986). However, some organs (e.g., adrenals and bone), commonly develop deposits, other organs (e.g., skin and lungs), occasionally develop deposits, whilst others (e.g., spleen and intestines), never develop deposits. This effect is reproducible with different tumours and appears to be a feature of the behaviour of the recipient tissue rather than the tumour (Murphy *et al.*, 1986). The resistance of the colon to growth of these experimental tumours is paralleled by the clinical observation that the large bowel is a rare site for bloodborne secondary deposits from primary malignancies elsewhere in the body.

Trauma to a tissue is known to enhance the ability of that tissue to support growth of tumour either from locally implanted cells (Jones & Rous, 1914), or from cells that reached the site of injury via the circulation (Robinson & Hoppe, 1962; Alexander & Altemeier, 1964; Fisher & Fisher, 1965).

Following intracardiac injection of the two tumours used in this investigation, Murphy *et al.* (1988) found that growth occurred much more readily in healing laparotomy wounds than in the surrounding normal skeletal muscle. There have been no studies to investigate the effect of surgical trauma on the ability of the large bowel to support growth of tumour cells delivered by the circulation. The aims of this investigation were firstly to determine whether surgical trauma to the colon would enhance its ability to support growth of bloodborne cancer cells; and secondly, if enhancement did take place, to determine at which stage in the healing process the enhancement is at a maximum.

### Materials and methods

### *Animals*

These were syngeneic hooded Lister rats, obtained initially from the Chester Beatty Institute and then maintained as an inbred line in Southampton. Both males (weight 200–300 g) and females (weight 150–250 g) were used.

### *Tumours*

Two tumours syngeneic for the hooded Lister rat were used. These were the MC28 sarcoma used for the majority of the experiments, and the OES5 breast carcinoma. MC28 is a methylcholanthrene-induced sarcoma and OES5 is an oestrogen-induced breast carcinoma (Senior *et al.*, 1985). Both tumours were maintained by subcutaneous passage, the MC28 every 14–21 days and the OES5 every 21–28 days. Growth of OES5 is oestrogen dependent and so all animals used with this tumour were females given oestrogen implants. The implant was made by heating and fusing together 80% Oestrone Gold Label (Aldrich Chemical Co.) and 20% cholesterol (Aldrich Chemical Co.) in a crucible.

### *Preparation of cell suspensions*

Tumours were removed from the flanks of passage animals, chopped finely with scissors, washed in Hank's balanced salt solution (Hank's BSS; Gibco) and then mechanically and enzymatically disaggregated using a mixture of protease 0.5 mg ml$^{-1}$ (Sigma No. P5647) and deoxyribonuclease 0.005 mg ml$^{-1}$ (Sigma No. D4638) in Hank's BSS and a magnetic stirrer for 45 min. After allowing large lumps to settle, the cell rich supernatant was pipetted off, spun down and washed twice with Hank's BSS. Viability counts were performed using trypan blue exclusion (Tennant, 1964) and dilutions were adjusted to give $10^5$ viable cells in 0.2 ml.

### *Radiolabelling of tumour cells*

OES5 cells could not be cultured and so no labelling experiments were performed with this tumour. MC28 cells were cultured in Dulbecco's Modified Eagle's Medium (DMEM; Gibco) plus 20% foetal calf serum (Gibco), 10,000 U ml$^{-1}$ penicillin and 10 mg ml$^{-1}$ streptomycin (Gibco) and nystatin 100 U ml$^{-1}$ (Sigma). Cells were suspended to give a final concentration of $4\times10^6$ viable cells per 5 ml culture medium. Six 25 cm$^2$ tissue culture flasks (NUNC) each received 5 ml of the suspension. Incubation was performed at 37°C in 5% $CO_2$ in humidified air. After 24 h the medium was changed and 5 $\mu$Ci 5$^{125}$I-iodo-2-deoxyuridine (Amersham International plc) was added to each flask together with 5 $\mu$M 5-fluoro-2-deoxyuridine (Sigma) to increase the incorporation of 5$^{125}$I-iodo-2-deoxyuridine. After a further 24 h the cells were removed by replacing the medium with Hank's BSS containing the mixture of protease and deoxyribonuclease. After 15 min, when the cells had detached, the suspension was washed twice in Hank's BSS and viability assessed using trypan blue exclusion. Dilution was adjusted to give $10^5$ viable cells in 0.2 ml. In order to assess radiolabelling, fixed cytospin preparations were autoradiographed using Ilford K2 emulsion and developed after 5 days using Ilford Contrast FF developer and Ilford

Correspondence: P. Alexander.
Received 9 October 1987; and in revised form, 1 February 1988.

Hypam fixer. Preparations were counterstained with Meyer's Haemalum. This demonstrated that 70% of tumour cells were labelled.

*Colonic anastomoses*

Under ether anaesthesia, the anterior abdominal wall was shaved and the abdomen opened through a midline incision. The left colon was delivered and transected with scissors, taking care not to injure the mesenteric arterial arcade. Any faeces in the immediate vicinity were removed but no formal attempt was made to prepare the bowel, no antibiotics were used and no dietary restrictions were imposed either pre or post operatively. The bowel was anastomosed using one layer of interrupted 6/0 silk sutures (Ethicon). The abdomen was then closed and the animals allowed to recover.

*Ileal anastomoses*

In addition to the colonic anastomosis, some animals also had an ileal anastomosis performed. The ileum was divided 2 cm proximal to the ileocaecal valve and anastomosed with one layer of interrupted 6/0 silk sutures.

*Tumour cell injections*

Under ether anaesthesia, the right internal carotid artery was exposed, isolated between ligatures and opened. A 0.6 mm outside diameter polythene cannula (Boro Labs) was passed proximally into the left ventricle. The correct position of the cannula was confirmed by noting the resistance of the aortic valve and the double wave form transmitted to the cannula. Cells ($10^5$) in 0.2 ml Hank's BSS were injected and flushed in with saline. The cannula was then removed, the carotid artery tied off and the skin closed. The animal was then allowed to recover.

*Relative timing of injection to anastomosis*

The day of the tumour cell injection was regarded as day 0. Colonic anastomoses were performed at intervals from day −14 to day +4 with respect to the tumour cell injection. Ileal anastomoses were performed on day −5 only.

*Post mortem examinations*

Animals were killed under ether anaesthesia on day +16 after tumour injection. The abdomen was opened and 1.5 cm of colon bearing the anastomosis was removed together with an equal length of normal large bowel lying immediately proximally. Where appropriate, 1.5 cm of ileum bearing the anastomosis was also removed. Specimens were opened, mounted on blotting card and fixed in 10% formalin. Longitudinal paraffin sections were made (3–5 for the anastomosis and 2–4 for the normal bowel), stained with haematoxalin and eosin and examined for tumour.

*Radiolabelled cell injections*

On day −5, a left colonic anastomosis was performed in two groups of 9 animals each. Each group received a separately prepared and labelled tumour cell injection. On day 0, $10^5$ radiolabelled MC28 cells were injected into the left ventricle and the animal killed 5 min later by injecting 20 mg pentobarbitone through the carotid cannula. The 1.5 cm of colon bearing the anastomosis and the adjacent 1.5 cm of proximal normal bowel were removed. These specimens plus a sample of $10^5$ cells were counted in a standard Wallac Decem series automatic well gamma-counter within 12 h of killing. No labelled cell injections were performed in animals with ileal anastomoses.

*Autoradiographs of colonic anastomoses*

Segments of bowel bearing an anastomosis were fixed in 10% formalin prior to gamma counting. After counting, the specimens were opened and paraffin embedded histological sections were made. These were then autoradiographed using Ilford K2 emulsion and developed after 5 days using Ilford Contrast FF developer and Ilford Hypam fixer. Specimens were counterstained with Meyer's Haemalum and mounted in DPX.

### Results

*Tumour cell injections*

Colonic anastomoses were performed prior to intracardiac injection of $10^5$ MC28 sarcoma cells on day −14 ($n=10$), day −8 ($n=10$), day −7 ($n=9$), day −5 ($n=17$), day −4 ($n=9$), day −2 ($n=14$), day −1 ($n=9$), 2 h prior ($n=15$); and after tumour injection at 1 h ($n=8$), on day +1 ($n=10$), and on day +4 ($n=5$). Tumour growth occurred only at anastomoses (Figure 1) and no tumour growth occurred in normal bowel in any animal in this series. Figure 2 shows the percentage of animals in each timing group bearing tumour at the anastomosis. Tumour growth occurred at the anastomotic site if anastomosis preceded tumour injection but no tumour growth occurred if surgery was performed after tumour injection. Maximum enhancement of tumour growth occurred at days −5 to −7, i.e., when the healing process had been in progress between 5 and 7 days. When the anastomosis preceded the tumour injection by 14 days, the enhancing effect of surgical trauma was mostly over. A small enhancement of tumour growth occurred if injection was performed within 2 h of anastomosis.

Injection of $10^5$ OES5 carcinoma cells 5 days after anastomosis resulted in tumour growth and the anastomosis in 2 of the 5 animals injected but not in normal bowel. No other timing was investigated with this tumour.

In 4 animals, both ileal and colonic anastomoses were performed 5 days prior to injection of $10^5$ MC28 cells. Tumour grew at all ileal anastomoses, and in greater quantity macroscopically than at the colonic anastomoses. No tumour growth occurred in normal ileum in any animal in this study.

**Figure 1** (a) Descending colon anastomosis with deposit of MC28 sarcoma (arrowed); (b) Photomicrograph of deposit of MC28 sarcoma at a colonic anastomosis (bar, 200 $\mu$m).

**Figure 2** Effect of timing of surgery on growth of MC28 sarcoma at colonic anastomoses. Day −14 *versus* day −8: $\chi^2=7.5$, $P<0.01$; Day −8 to day −2: n.s.; Day −2 *versus* day −1: $\chi^2=6.3$, $P<0.02$; Day −1 *versus* −2 h: n.s.; Day −2 *versus* −2 h: $\chi^2=4.14$, $P<0.05$; −2 h *versus* +1 h: n.s.; Day −1 plus −2 h *versus* +1 h plus day +1: $\chi^2=4.26$, $P<0.05$.

*Radiolabelled cell injections*

Two groups of 9 animals each were injected with $10^5$ labelled MC28 sarcoma cells in two separate experiments. Radiolabelling of cells in the first experiment was greater by a factor of four compared to the second experiment. There were variations in the counts per minute between the anastomoses of different animals and also the normal bowel between different animals (Tables I and II). The geometric means of the ratios of counts in anastomoses to normal bowel were calculated for experiment 1 and experiment 2, along with the 95% confidence intervals. There was approximately a 1.5–1.6 times greater trapping of cells in the anastomosis compared to the normal colon. As unity falls between the 95% confidence intervals for both experiments, this difference in trapping does not reach formal statistical significance. However, with the good agreement between the geometric mean ratio for both experiments and the fact that unity is only just within the 95% confidence intervals, it may well be that the difference in trapping in the anastomosis is a real phenomenon.

In the first injection series (Table I), $10^5$ cells resulted in an estimated 92 cells arresting in 1.5 cm of normal colon and 141 cells arresting in the 1.5 cm of colon bearing the anastomosis. In the second series (Table II), $10^5$ cells gave an estimated 72 cells in 1.5 cm of normal colon and 118 cells trapping in the 1.5 cm bearing the anastomosis. Figures from both series agree well, and, taking an average of both series, we calculate that from $10^5$ cells injected, 82 cells trap in 1.5 cm of normal colon and 130 in the 1.5 cm bearing the anastomosis. These 130 cells trapped at the anastomosis lead to an average of three tumour nodules per anastomosis, i.e., there is a chance of 1:43 that a cell arriving at an anastomosis will lead to a deposit.

Approximately 82 cells arrive in 1.5 cm of normal colon, i.e., $\sim 0.8\times10^3$ cells or 0.8% of the total injected arrive in the entire 15 cm of large bowel. This is of the same order as results reported by Murphy *et al.* (1986) who found 1.7% of injected MC28 sarcoma cells trapping in the colon. No tumours are induced in normal colon. Therefore, in normal colon, a trapped cell has a less than $1:0.8\times10^3$ chance of forming a deposit. This value is in fact a considerable overestimate. Murphy *et al.* (1986) showed that even after intracardiac injection of $5\times10^6$ cancer cells, calculated to result in the trapping of $4\times10^4$ cancer cells in the colon, no deposits arose. Accordingly, the probability of an MC28 sarcoma cell producing a metastasis in normal colon is less than $1:4\times10^4$. The autoradiographs of the histological sections of anastomoses after intracardiac injection of labelled tumour cells did not demonstrate any grouping or re-distribution of tumour cells in the region of the healing anastomosis.

### Discussion

Jones & Rous (1914) demonstrated that injuring the mouse peritoneum with either Kieslguhr (to give a generalised injury) or with glass rods (to give a localised injury) enhanced growth of intraperitoneally injected tumour cells, the enhancement being restricted to the area of injury in the case of the glass rods. Other workers have demonstrated limb trauma (Fisher *et al.*, 1967), hepatic trauma (Fisher & Fisher, 1965), and splenic trauma (Alexander & Altemeier, 1964) to enhance development of metastases in these organs from bloodborne tumour cells. The normal colon and ileum are refractory to growth of bloodborne tumour cells used in this study (Murphy *et al.*, 1986) and the colon and ileum are clinically very rare sites for secondary deposits from tumours elsewhere in the body. However, Alexander & Altemeier (1964) had some tumour deposits in the intestines after intra-

**Table I** Arrest of radiolabelled tumour cells: Experiment 1

| *Animal number* | *Normal bowel (1.5 cm) cpm* | *Anastomosis (1.5 cm) cpm* | *Ratio anastomosis/normal* |
|---|---|---|---|
| 1 | 156 | 315 | 2.02 |
| 2 | 362 | 410 | 1.13 |
| 3 | 183 | 191 | 1.04 |
| 4 | 20 | 158 | 7.90 |
| 5 | 229 | 197 | 0.86 |
| 6 | 2,031 | 905 | 0.45 |
| 7 | 246 | 391 | 1.59 |
| 8 | 43 | 72 | 1.67 |
| 9 | 243 | 563 | 2.32 |
| Geometric mean cpm | 184 | 281 | 1.53 |
| 95% confidence intervals | | | 0.83–2.81 |
| Estimated number of cells trapped | 92 | 141 | |

$10^5$ cells gave 203,536 cpm, i.e. 1 cell gave 2 cpm. Each sample was counted for 10 min and the background count of 43 $min^{-1}$ was automatically subtracted.

**Table II** Arrest of radiolabelled tumour cells: Experiment 2

| *Animal number* | *Normal bowel (1.5 cm) cpm* | *Anastomosis (1.5 cm) cpm* | *Ratio anastomosis/normal* |
|---|---|---|---|
| 1 | 8 | 38 | 4.75 |
| 2 | 18 | 38 | 2.11 |
| 3 | 42 | 41 | 0.98 |
| 4 | 173 | 95 | 0.55 |
| 5 | 40 | 139 | 3.48 |
| 6 | 58 | 81 | 1.40 |
| 7 | 36 | 51 | 1.42 |
| 8 | ·39 | 38 | 0.97 |
| 9 | 33 | 73 | 2.21 |
| Geometric mean cpm | 36 | 59 | 1.60 |
| 95% confidence intervals | | | 0.97–2.65 |
| Estimated number of cells trapped | 72 | 118 | |

$10^5$ cells gave 49,334 cpm, i.e., 2 cells gave 1 cpm. Each sample was counted for 10 min and the background count of 41 $min^{-1}$ was automatically subtracted.

aortic injection of V2 carcinoma cells in the rabbit, and handling the bowel increased the number of animals with deposits. No previous reports exist of the effect of surgical transection and anastomosis on enhancing tumour growth in large or small bowel. Cohn (1967) showed that Brown Pearce tumour cells introduced into the lumen of the rabbit bowel could implant on a colonic suture line. However, this gives little indication of the enhancement of tumour growth caused by trauma to the colon as the presence of the colonic suture line may merely allow large numbers of cells to gain access to the tissues rather than *per se* enhance tumour growth.

From the results of this study it is clear that surgical trauma enhances tumour growth from bloodborne cancer cells in large bowel when the trauma precedes the tumour injection. Maximum enhancement appears to occur when the healing process has progressed between 2 to 8 days with a peak between 5 and 7 days. A smaller peak occurs if cells reach the anastomosis within 2 h of its formation. A previous study (Alexander & Altemeier, 1964), utilising chemical trauma to the spleen, showed a maximum enhancement when trauma preceded tumour injection by 2 to 5 days, the effect then subsiding but still being present up to 37 days. In the present study, enhancement is largely over when the trauma precedes the tumour injection by 14 days. It is noteworthy that transecting the bowel after the cells have reached the tissue does not cause detectable enhancement of tumour growth, even though tumour cells are known to remain viable for up to 24 h after trapping in tissues (Murphy *et al.*, 1988). Enhanced tumour growth at a healing colonic anastomosis is not restricted to one tumour (MC28) as the OES5 carcinoma also grows at colonic anastomoses. Tumour growth is also enhanced by ileal anastomosis, although the effect of timing on enhancement has not been studied.

Fisher *et al.* (1967) showed that trauma to a hind limb increased the trapping of arterially injected tumour cells by 1.9–3 times for mechanical trauma, 3.6–9.8 times for chemical trauma and 1.6–2.5 times for surgical incision and suture. The figures of Fisher *et al.* (1967) for increased cell trapping after surgery agree well with the present study where cell trapping was increased by 1.5–1.6 times in the 1.5 cm of colon bearing the anastomosis, compared to an equal length of normal colon. However, a cell arriving at the anastomosis stands a 1:43 chance of forming a deposit whereas for a cell arriving in normal colon the chance is less than $1{:}4 \times 10^4$. There is approximately a 1,000 fold increase in the soil effect in anastomosed colon compared to non operated bowel. To express a 'soil effect' numerically as a probability that a single cell will cause a deposit requires that following intracardiac injection, the cells are distributed singly within the tissues. This was shown to be the case for normal tissue by Murphy *et al.* (1986; 1988) who found that following intracardiac injection of single cell suspensions, the cells distributed in the organs singly and randomly. Individual cells were far apart and there was no possibility that the tumour formed as the concerted action of several cancer cells. We have no direct evidence of the distribution of arrested cells in the 1.5 cm of anastomosed colon. However, as cell trapping is increased by only 1.5–1.6 times in the anastomosed segment, one would need to postulate cells being directed away from the normal areas within that 1.5 cm to the actual healing area. Also, if a change in haemodynamics and new vessel formation is leading to a redistribution of cells, it is unlikely that this effect would persist throughout the entire period of tumour growth enhancement, i.e., from within 2 h after the surgery through to 14 days after the surgery. The haemodynamics and the amount of new vessel formation is very different between days 2 and 8 of the healing process, which is a period when enhancement of tumour growth is maximal. Lastly, autoradiographs of histological sections of the anastomoses did not demonstrate any grouping of tumour cells at the anastomotic line, although total numbers of cells arrested would have been small and hence difficult to detect unless grouping at the anastomosis had been particularly marked. We therefore feel that the observed enhancement of tumour growth is due to the influence of the healing process.

The implications of these observations are twofold. Firstly this method provides a model for studying the effect of the healing process on metastatic growth. The advantages of this model are that no tumour deposition occurs unless the bowel is traumatised, eliminating any background; and that the trauma is easily and consistently reproduced. Secondly this enhancement of tumour growth in the healing colonic anastomosis may have importance in local recurrence of colorectal cancer. Arterial delivery of experimental tumour cells to a colonic anastomosis is not a model for local recurrence of a human cancer. Rather, it demonstrates that the healing process enhances tumour growth and indicates the order of magnitude of this enhancement. Although enhancement is most marked when healing has progressed between 2 and 8 days, there is still enhancement of tumour growth in colonic anastomoses in the first few hours following surgery, the time when cells spilled during surgery would encounter the surrounding healing tissues. Spillage of exfoliated malignant cells from the lumen of the bowel is held to be one cause of local recurrence (Umpleby *et al.*, 1984; Skipper *et al.*, 1987) and viable cells spilled into this enhanced environment for tumour growth may be more likely to implant and form a recurrence.

We would like to thank Mr R. Lee of the University Histology Department for his meticulous care in preparing the sections and we are grateful to the staff of the Department of Nuclear Medicine for performing the gamma-counting. Thanks are also due to Mr T. Richards and his technical staff. The work was supported by a generous grant from the Cancer Research Campaign. D.S. is a Cancer Research Campaign Fellow.

### References

ALEXANDER, J.W. & ALTEMEIER, W.A. (1964). Susceptibility of injured tissue to haematogenous metastases: An experimental study. *Ann. Surg.*, **159**, 933.

COHN, I. (1967). Implantation in cancer of the colon. *Surg. Gynecol. Obstet.*, **124**, 501.

FISHER, B., FISHER, E.R. & FEDUSKA, N. (1967). Trauma and the localisation of tumour cells. *Cancer*, **20**, 23.

FISHER, E.R. & FISHER, B. (1965). Experimental study of factors influencing development of hepatic metastases from circulating tumour cells. *Acta Cytologica*, **9**, 146.

JONES, F.S. & ROUS, P. (1914). On the cause of the localisation of secondary tumours at points of injury. *J. Exp. Med.*, **20**, 404.

MURPHY, P., ALEXANDER, P., KIRKHAM, N., FLEMING, J. & TAYLOR, I. (1986). Pattern of spread of bloodborne tumour. *Br. J. Surg.*, **73**, 829.

MURPHY, P., ALEXANDER, P., SENIOR, P.V., FLEMING, J., KIRKHAM, N. & TAYLOR, I. (1988). Mechanisms of organ selective tumour growth by bloodborne cancer cells. *Br. J. Cancer*, **57**, 19.

ROBINSON, K.P. & HOPPE, E. (1962). The development of bloodborne metastases: Effect of local trauma and ischaemia. *Arch. Surg.*, **85**, 720.

SENIOR, P.V., MURPHY, P. & ALEXANDER, P. (1985). Oestrogen dependent rat mammary carcinoma as a model for dormant metastases. In *Treatment of Metastases: Problems and Prospects*, Hellman, K. & Eccles, S.A. (eds) p. 113. Taylor and Francis: London.

SKIPPER, D., COOPER, A.J., MARSTON, J.E. & TAYLOR, I. (1987). Exfoliated cells and *in vitro* growth in colorectal cancer. *Br. J. Surg.*, **74**, 1049.

TENNANT, J.R. (1964). Evaluation of the trypan blue technique for determination of cell viability. *Transplantation*, **2**, 685.

UMPLEBY, H.C., FERMOR, B., SYMES, M.O. & WILLIAMSON, R.C.N. (1984). Viability of exfoliated colorectal carcinoma cells. *Br. J. Surg.*, **71**, 659.